# Cost-effectiveness analysis of bridge-to-transplantation temporary mechanical circulatory support versus non-bridged heart transplantation

**DOI:** 10.3389/fpubh.2025.1687327

**Published:** 2026-01-08

**Authors:** Lei Yin, Xiuli An, Bin Yang, Zhiming Zhou, Yuhan Li, Chunhui Wang, Soumitra S. Bhuyan, Yafei Si, Pengfei Wang, Jianping Hu, Wanliang Zhang, Clifford Silver Tarimo, Jingming Wei, Quanman Li, Zhanlei Shen, Qingyong Lu, Yudong Miao, Xinran Li

**Affiliations:** 1 The 7th People’s Hospital of Zhengzhou, Zhengzhou, Henan, China; 2 Edward J. Bloustein School of Planning & Public Policy Rutgers, The State University of New Jersey, New Brunswick, NJ, United States; 3 Melbourne School of Population and Global Health, The University of Melbourne, Melbourne, VIC, Australia; 4 Department of Biomedical Engineering, Southern University of Science and Technology, Shenzhen, Guangdong, China; 5 Henan Medical Communication and Project Forward Center, Zhengzhou, Henan, China; 6 Department of Health Management, College of Public Health, Zhengzhou University, Zhengzhou, Henan, China; 7 Department of Science and Laboratory Technology, Dar es salaam Institute of Technology, Dar es Salaam, Tanzania

**Keywords:** heart failure, heart transplantation, temporary mechanical circulatory support, cost-effectiveness, Markov model, health economics

## Abstract

**Objectives:**

Among patients undergoing heart transplantation, BTT-tMCS therapy demonstrates superior clinical efficacy compared to nonbridged HTx; however, its cost-effectiveness among Chinese patients remain uncertain. To evaluate the cost-effectiveness of BTT-tMCS therapy compared with nonbridged HTx among transplant-eligible Chinese patients from the healthcare payer’s perspective.

**Methods:**

The cost-effectiveness analysis for this economic evaluation was conducted at a qualified heart transplantation center in Central China. Participants included patients admitted since 2018 who underwent either BTT-tMCS or nonbridged HTx, identified retrospectively from local electronic medical records and supplemented by corresponding questionnaires. Propensity score matching (PSM) was employed to obtain a homogeneous group of HTx patients to generate model input parameters. A Markov model simulates lifetime disease progression and associated costs for HTx patients, with monetary values standardized to 2023. Sensitivity analyses were performed to test the internal validity of the model’s conclusions. The model evaluated two competing treatment strategies: BTT-tMCS and standard care. Lifetime health care cost and quality-adjusted life-years (QALYs) of the simulated cohort.

**Results:**

PSM identified 40 matched subjects who met the predefined criteria, creating a homogeneous cohort for analysis. Compared to nonbridged HTx, BTT-tMCS yielded higher lifetime incremental costs (510,361 RMB) and effects (5.88 QALYs), resulting in an ICER of 28966.88 RMB/QALY. Sensitivity analyses demonstrated the robustness of these findings, with a 90% probability of cost-effectiveness achieved at a willingness-to-pay threshold exceeding 80422.20 RMB/QALY.

**Conclusion:**

In this economic evaluation study, BTT-tMCS therapy was more likely to be cost-effective compared with non-bridged HTx.

## Introduction

Heart transplantation (HTx) is the definitive treatment for end-stage heart failure, significantly improving patient survival and quality of life (1–3). However, persistent donor shortages result in long waiting times, during which many patients deteriorate or die (4, 5). Temporary mechanical circulatory support (tMCS) has emerged as a critical bridge-to-transplantation strategy, stabilizing patients and improving transplant eligibility (6).

Devices such as extracorporeal membrane oxygenation (ECMO), intra-aortic balloon pumps (IABP), and ventricular assist devices (VAD) are increasingly used to maintain cardiac function and preserve end-organ perfusion in transplant candidates (7–9). International registries, including the United Network for Organ Sharing (UNOS) and the International Society for Heart and Lung Transplantation (ISHLT), report improved outcomes among patients bridged with tMCS, including reduced pre-transplant mortality and shorter waitlist times (10–12). Although device-related complications may temporarily impair functional status, the health-related quality of life of patients who previously received temporary mechanical circulatory support (tMCS) remains generally well-preserved after transplantation, with a mortality rate comparable to or even higher than that of the non-tMCS group (13–15). Notably, emerging evidence reveals important sex differences: women experience higher rates of adverse events with left ventricular assist devices and ECMO, yet may exhibit better long-term survival after heart transplantation than men (16).

Despite growing clinical evidence, economic evaluations of BTT-tMCS remain limited-particularly in low- and middle-income countries (LMICs), where cost-effectiveness is a key determinant of clinical adoption (17, 18). In China, where HTx volume is rising, healthcare resources are limited, and tMCS remains costly, rigorous economic evaluation is essential for supporting clinical and policy decisions. However, most existing studies are based on Western data and healthcare systems (19), limiting their relevance to the Chinese context.

To address this gap, we conducted a cost-effectiveness analysis of BTT-tMCS compared with nonbridged HTx at a Chinese transplant center, using a Markov model informed by local clinical and economic data. This study aimed to generate empirical evidence to guide clinical decisions and optimize the use of healthcare resources in transplantation settings, including considerations for prioritizing BTT-tMCS in reimbursement policies or clinical guidelines. The findings aim to support evidence-based allocation of limited healthcare resources and promote more equitable access to life-saving interventions.

## Methods

### Survey design and participants

Hospital Information System (HIS) data from a central China heart transplant center were analyzed retrospectively. Case records of heart transplant recipients admitted between 2018 and August 2024 were extracted during a data collection period spanning July and August 2024. The extracted data included: (1) patient demographics (sex, age, etc.); (2) admission characteristics (primary diagnosis, underlying diseases, etc.); and (3) hospitalization costs (total and average costs).

Trained personnel conducted phone interviews using structured questionnaires to collect data on socio-demographic characteristics and healthy quality of life. Rigorous training, emphasizing standardized interviewing techniques, including scripted questions and avoiding leading questions, was provided to minimize interviewer bias. A pilot test was conducted to refine the questionnaire and optimize training procedures. Experienced researchers oversaw data collection to ensure study protocol adherence and data quality.

Inclusion criteria for the current study were defined as patients who underwent HTx at the designated hospital and were registered within the HIS. Conversely, exclusion criteria encompassed: (1) instances of missing, anomalous, or logically inconsistent data; (2) incomplete questionnaire responses; and (3) patients who died. Propensity Score Matching (PSM) was used to obtain homogenized heart transplant patients with bridge-to-transplantation temporary mechanical circulatory support (BTT-tMCS) (Group A) and non-bridged heart transplant patients (HTx) (Group B) for the study, removing participants missing important outcome variables (costs and QALYs).

All participants provide informed consent prior to data collection and all data is kept strictly confidential. After the data was anonymized, the study received ethical approval from the ethics Committee of the hospital (Registration number: 2024-0619). The inclusion–exclusion criteria are depicted in Supplementary Figure S1.

### Model structure

We constructed a Markov model to assess the clinical benefits and economic impact of BTT-tMCS therapy and nonbridged HTx as treatment strategies. The model estimated the outcomes for patients undergoing 2 treatment strategies: implantation of a BTT-tMCS with subsequent HTx and nonbridged HTx. In addition, we performed gender subgroup analyses of the two treatment strategies. Model outputs were represented in terms of cost, quality-adjusted life years (QALYs), and incremental cost-effectiveness ratio (ICER). The analysis was conducted using TreeAge Pro version 2022 (TreeAge Software).

The Markov model was used to project the outcomes and costs of the 2 treatment pathways (BTT-VAD group and nonbridged HTx group). The HTx group started from the survival state and could progress to implant MCS, or death. The BTT-tMCS group started from the survival state and could progress to death. After receiving HTx or a tMCS, patients could develop complications. The model schematic is presented in Supplementary Figure S2.

The analytic time horizon was set 10 years, with a one-year interval defined as a cycle (20). The assumptions of the model, made in concordance with expert opinion, were as follows: (1) All the patients were heart transplant recipients; a patient undergoes a tMCS implantation and subsequent heart transplantation, who without HTx waiting state. (2) Postoperative complications are also considered survival state. (3) A patient in a given state could make only a single state transition during a cycle; transitions were considered to occur at the midpoint of each cycle. (4) The pre-tMCS or pre-HTx mortality in patients undergoing implantation was considered null. (5) A second MCS was not considered an option in this model. (6) The development of any post-tMCS or post-transplantation state was assumed to be exclusive, which meant that patients could not develop 2 states simultaneously.

### Parameter definition

#### Transfer probabilities

The parameters mainly comprise three categories: probabilities, costs, and utilities. In this study, the outcome probabilities were estimated using cohort data considered representative of the target population. Mortality rates and in-hospital renal failure requiring dialysis rates for patients with and without pre-transplant temporary mechanical circulatory support were estimated from a cohort of 6,892 orthotopic heart transplant recipients (15). Similarly, estimating the transition probability of in-hospital based on retrospective cohort data from a single large transplant center (21). The annual incidences of infection were estimated from data on 1,030 heart transplant recipients (22). The transition probabilities of renal failure per cycle were calculated from the renal failure requiring dialysis rates of 14,635 orthotopic heart transplant recipients (23). The probabilities of postoperative MCS reimplantation and survival were inferred and summarized from the data of 1,417 heart transplant cases (24). The probabilities in the Markov model were consistent with the data describing these events.

Due to the limited availability of high-quality domestic cohort data related to post-transplantation transition events in China, several transition probabilities used in this study were derived from well-established international cohorts. While these sources originate outside of China, they were selected based on methodological rigor, large sample size, and clinical relevance. Furthermore, the key transition probabilities reflect general post-transplant disease progression patterns (e.g., infection, renal failure, or mortality), which are less influenced by ethnicity and more related to clinical procedure and organ transplantation physiology. Extensive one-way and probabilistic sensitivity analyses were conducted to account for uncertainties associated with cross-population data application, consistently confirming the model’s reliability within plausible ranges. Supplementary Figure S4 presents the state probability analysis results derived from the Markov cohort model, demonstrating close alignment with real-world survival data from China. Therefore, while acknowledging the limitation of reliance on international data, the included parameters are deemed reasonably representative for use in Chinese transplant-eligible patients.

#### Costs

Cost data were analyzed from a healthcare payer perspective, encompassing the initial hospitalization costs for heart transplantation surgery and the subsequent annual costs required to maintain patients in each defined health state within the Markov model. The hospitalization expenses of patients were extracted from the HIS. The costs of hospitalized patients originated from the following aspects: (1) diagnostic tests (X-rays, ultrasounds, blood tests, microbiology and pathology tests); (2) drug and pharmacy labor costs; (3) surgical fees; (4) device costs for patients who received tMCS implantation. The medical costs per cycle were estimated based on the literature related to the economic burden of diseases and the actual situation in China (22). Costs from previous years were inflated to and expressed in the 2023 Chinese RMB using the consumer price index from Statistics China (25).

#### Effectiveness

Effectiveness was measured in quality-adjusted life-years (QALYs), which accounts for health-related quality of life and length of life. QALY was calculated based on the EQ-5D-5L at 12 months after HTx, which consists of the dimensions “mobility,” “self-care,” “usual activities,” “pain/discomfort” and “anxiety/depression” (26). Participants rated their health on a scale from “no problems” (1) to “extreme problems” (5) across multiple dimensions. Combining these responses generated an individual health state, with 3,125 possible health states in total. A health state of “11,111” represented the best possible health, while “55,555” indicated the worst. Health states were mapped to EQ-5D-5L index scores using Chinese preference-based value sets, where a score of 0 represented death and 1 indicated perfect health. Utility estimates for patients receiving BTT-tMCS and nonbridged HTx were derived from EQ-5D-5L scores, calculated based on questionnaire responses from heart transplant recipients (Supplementary Table S1). A half-cycle correction of costs and effects was carried out. We applied an annual discount of 5%.

### Sensitivity analysis

Sensitivity analyses were conducted over a 10-year time period, examining the impact of uncertainty in parameters. One-way deterministic sensitivity analyses were performed by individually varying each probability parameter (as detailed in Table 1) within its 95% confidence interval. The cost data were modified using the values of quartiles 1 and 3, detailed in Table 2. An incremental net monetary benefit (INMB) Tornado diagram visually represented the outcomes of the analysis. Subsequently, a probabilistic sensitivity analysis was conducted to assess the impact of parameter uncertainty on the cost-effectiveness results. In the PSA, probability and utility parameters were assigned beta distributions, and cost parameters were assigned gamma distributions. A Monte Carlo simulation with 1,000 iterations was performed, randomly sampling values from these distributions to generate 1,000 paired estimates of costs and effectiveness (measured in quality-adjusted life years, QALYs) for bridge-to-transplant with temporary mechanical circulatory support (BTT-tMCS) versus non-bridged heart transplantation (HTx). The simulation results were plotted onto a cost-effectiveness plane and used to generate a cost-effectiveness acceptability curve (CEAC), which illustrated the probability of BTT-tMCS being cost-effective compared to non-bridged HTx across a range of willingness-to-pay (WTP) thresholds.

**Table 1:** Model input parameters of transition probabilities.

From	To	Probabilities (95% confidence interval)	Distribution
BTT-tMCS			
Survival	Dead	0.046 (0.039–0.053)	Beta
Survival	Infection	0.366 (0.312–0.419)	Beta
	Infection (>1 annually)	0.362 (0.333–0.392)	Beta
Survival	Renal failure	0.426 (0.410–0.442)	Beta
	Renal failure (>1 annually)	0.074 (0.070–0.078)	Beta
HTx			
Survival	Dead	0.050 (0.043–0.057)	Beta
Survival	Infection	0.269 (0.220–0.318)	Beta
	Infection (>1 annually)	0.362 (0.333–0.392)	Beta
Survival	Renal failure	0.302 (0.287–0.317)	Beta
	Renal failure (>1 annually)	0.074 (0.070–0.078)	Beta
Survival	MCS	0.037 (0.027–0.047)	Beta
Implant MCS			
MCS	Survival	0.330 (0.306–0.354)	Beta

Probability of cost-effectiveness depends on the willingness to pay.

**Table 2:** Patient demographics of included studies.

Variables	Before PSM				After PSM			
	Total (%)	tMCS (%)	HTx (%)	*p*-value	Total (%)	tMCS (%)	HTx (%)	*p*-value
Total	134 (100)	66 (49.25)	68 (50.75)		80 (100)	40 (50.00)	40 (50.00)	
Sex				0.167				0.034
Male	114 (85.07)	59 (89.39)	55 (80.88)		67 (83.75)	37 (92.50)	30 (75.00)	
Female	20 (14.93)	7 (10.61)	13 (19.12)		13 (16.25)	3 (7.50)	10 (25.00)	
Age, years				0.034				0.412
≤60	102 (76.12)	45 (68.18)	57 (83.82)		63 (78.75)	33 (82.50)	30 (75.00)	
>60	32 (23.88)	21 (31.82)	11 (16.18)		17 (21.25)	7 (17.50)	10 (25.00)	
Education levels				0.220				0.400
Primary and below	28 (20.90)	10 (15.15)	18 (26.47)		19 (23.75)	7 (17.50)	12 (30.00)	
Junior and senior high school	84 (62.69)	43 (65.15)	41 (60.29)		49 (61.25)	26 (65.00)	23 (57.50)	
College or above	22 (16.42)	13 (19.70)	9 (13.24)		12 (15.00)	7 (17.50)	5 (12.50)	
Marry				0.763				0.775
Married	107 (79.85)	52 (78.79)	55 (80.88)		65 (81.25)	32 (80.00)	33 (82.50)	
Others	27 (20.15)	14 (21.21)	13 (19.12)		15 (18.75)	8 (20.00)	7 (17.50)	
Occupation				0.625				0.217
Manual worker	42 (31.34)	22 (33.33)	20 (29.41)		57 (71.25)	26 (65.00)	31 (77.50)	
Non-manual worker	92 (68.66)	44 (66.67)	48 (70.59)		23 (28.75)	14 (35.00)	9 (22.50)	
Medical insurance type				0.245				0.785
Employee basic medical insurance	27 (20.15)	16 (24.24)	11 (16.18)		17 (21.25)	9 (22.50)	8 (20.00)	
Resident basic medical insurance	107 (79.85)	50 (75.76)	57 (83.82)		63 (78.75)	31 (77.50)	32 (80.00)	
Blood type				0.834				0.636
A	40 (29.85)	19 (28.79)	21 (30.88)		24 (30.00)	10 (25.00)	14 (35.00)	
B	46 (34.33)	25 (37.88)	21 (30.88)		27 (33.75)	16 (40.00)	11 (27.50)	
AB	12 (8.96)	5 (7.58)	7 (10.29)		9 (11.25)	4 (10.00)	5 (12.50)	
O	36 (26.87)	17 (25.76)	19 (27.94)		20 (25.00)	10 (25.00)	10 (25.00)	
NYHA				0.063				0.077
III	15 (11.19)	4 (6.06)	11 (16.18)		9 (11.25)	2 (5.00)	7 (17.50)	
IV	119 (88.81)	62 (93.94)	57 (83.82)		71 (88.75)	38 (95.00)	33 (82.50)	
Primary disease				0.032				0.998
Dilated -cardiomyopathy	79 (58.96)	45 (68.18)	34 (50.00)		48 (60.00)	24 (60.00)	24 (60.00)	
Others	55 (41.04)	21 (31.82)	34 (50.00)		32 (40.00)	16 (40.00)	16 (40.00)	
CI				0.664				0.962
	1.98 (1.70–2.39)	1.98 (1.67–2.43)	1.98 (1.80–2.33)		2.00 (1.75–2.47)	2.00 (1.80–2.41)	2.02 (1.68–2.52)	
PAWP				0.021				0.567
	15.75 (11.29–21.33)	14.59 (10.03–19.68)	17.91 (13.33–24.20)		14.96 (10.81–19.94)	14.00 (10.36–19.88)	15.31 (11.30–20.62)	
PVR				0.187				0.421
	2.00 (0.97–2.79)	2.24 (1.01–2.87)	1.70 (0.77–2.73)		2.24 (0.97–3.07)	2.30 (1.09–2.86)	1.89 (0.69–3.37)	
mPAP				0.016				0.889
	25.12 (20.65–30.48)	26.79 (22.67–31.52)	23.14 (18.07–28.06)		26.12 (20.96–31.38)	26.14 (21.09–30.69)	25.93 (20.96–32.94)	
TPG				0.524				0.835
	9.00 (7.00–12.00)	9.00 (7.00–12.00)	8.50 (6.00–13.00)		9.50 (7.00–13.00)	9.50 (7.00–12.50)	9.50 (6.00–13.00)	
CMV				0.846				0.745
No	117 (87.31)	58 (87.88)	59 (86.76)		69 (86.25)	34 (85.00)	35 (87.50)	
Yes	17 (12.69)	8 (12.12)	9 (13.24)		11 (13.75)	6 (15.00)	5 (12.50)	
Preoperative infection				0.447				0.531
No	119 (88.81)	60 (90.91)	59 (86.76)		68 (85.00)	35 (87.50)	33 (87.50)	
Yes	15 (12.69)	6 (9.09)	9 (13.24)		12 (15.00)	5 (12.50)	7 (12.50)	

PSM, propensity score matching; tMCS, bridge-to-transplantation temporary mechanical circulatory support; HTx, non-bridged heart transplantation; CI, cardiac index; PAWP, pulmonary artery wedge pressure; PVR, pulmonary vascular resistance; mPAP, mean pulmonary artery pressure; TPG, transpulmonary gradient, CMV, cytomegalovirus infection.

Following World Health Organization (WHO) recommendations (27), the WTP threshold was set at one to three times the per capita gross domestic product (GDP). In 2023, China’s per capita GDP was 89,358 RMB, as reported by the National Bureau of Statistics of China (28). Accordingly, the WTP thresholds applied in this study ranged from 0 RMB to 268,074 RMB per QALY gained.

## Results

### Patient demographics of included studies

Among the 134 HTx patients included, 66 patients (49.25%) were treated with BTT-tMCS, and 79 patients (58.96%) had dilated cardiomyopathy. There were 32 older HTx patients aged 60 and above (23.88%). The mean values of PAWP and mPAP were 15.75 and 25.12, respectively. Significant differences were observed between the two groups across several variables (all *p* < 0.05). Following PSM analysis, 80 participants were successfully matched from the original 134 patients. After matching for age, primary disease, PAWP, and mPAP, no statistically significant differences were observed between the BTT-tMCS and nonbridged HTx groups across covariates, except for gender (*p* > 0.05). The PSM balance test results are presented in Supplementary Figure S3. (The PSM balance test of tMCS and non-bridged HTx samples is shown in Supplementary Figure S3).

### Cost-effectiveness analysis

The results from the present analysis projected improvements in clinical outcomes with increased costs in patients undergoing a tMCS implantation versus nonbridged HTx patients. Table 3 shows the estimated values of the cost-effectiveness analysis at different time points. At a time horizon of 10 years, tMCS therapy increased quality-adjusted life years at increased cost relative to heart transplant recipients. Compared to nonbridged HTx patients, the cost of BTT-tMCS was 510,361 RMB per person, with an increase of 37,156 RMB per person. The average QALYs obtained per capita was 5.88, which was 1.28 QALYs more. Each extra QALY obtained per capita cost 28966.88 RMB.

**Table 3:** Cost-effectiveness results.

Time	Model outcome	tMCS	HTx	tMCS-HTx
1 year	Cost*(RMB)	351,399	335,266	16,133
	QALYs	1.25	1.04	0.22
	ICER (RMB/QALY)			74230.37
5 years	Cost*(RMB)	445,938	421,189	24,748
	QALYs	3.90	3.17	0.73
	ICER (RMB/QALY)			33942.94
10 years	Cost*(RMB)	510,361	473,205	37,156
	QALYs	5.88	4.60	1.28
	ICER (RMB/QALY)			28966.88

### Subgroup analysis

Subgroup analysis stratified by sex revealed that BTT-tMCS therapy was more cost-effective than nonbridged HTx in both male and female patients. In the male cohort, BTT-tMCS resulted in a gain of 0.97 QALYs and an increase in costs by 118,624 RMB. Similarly, in the female cohort, BTT-tMCS was associated with incremental costs of 287,863 RMB and a gain of 1.35 QALYs (see Table 4).

**Table 4:** Subgroup analysis results.

Therapy	Model outcome	Male	Female	Male–Female
tMCS	Cost*(RMB)	435,477	538,674	−103,196
	QALYs	3.92	3.88	0.04
	ICER (RMB/QALY)			−2659634.77 (male dominated)
HTx	Cost*(RMB)	317,213	250,811	66,402
	QALYs	2.95	2.53	0.42
	ICER (RMB/QALY)			159050.30 (male dominated)
tMCS-HTx	Cost*(RMB)	118,624	287,863	/
	QALYs	0.97	1.35	/
	ICER (RMB/QALY)	122028.68 (BTT-tMCS dominated)	213573.43 (BTT-tMCS dominated)	/

### Sensitivity analysis

**Figure 1:**
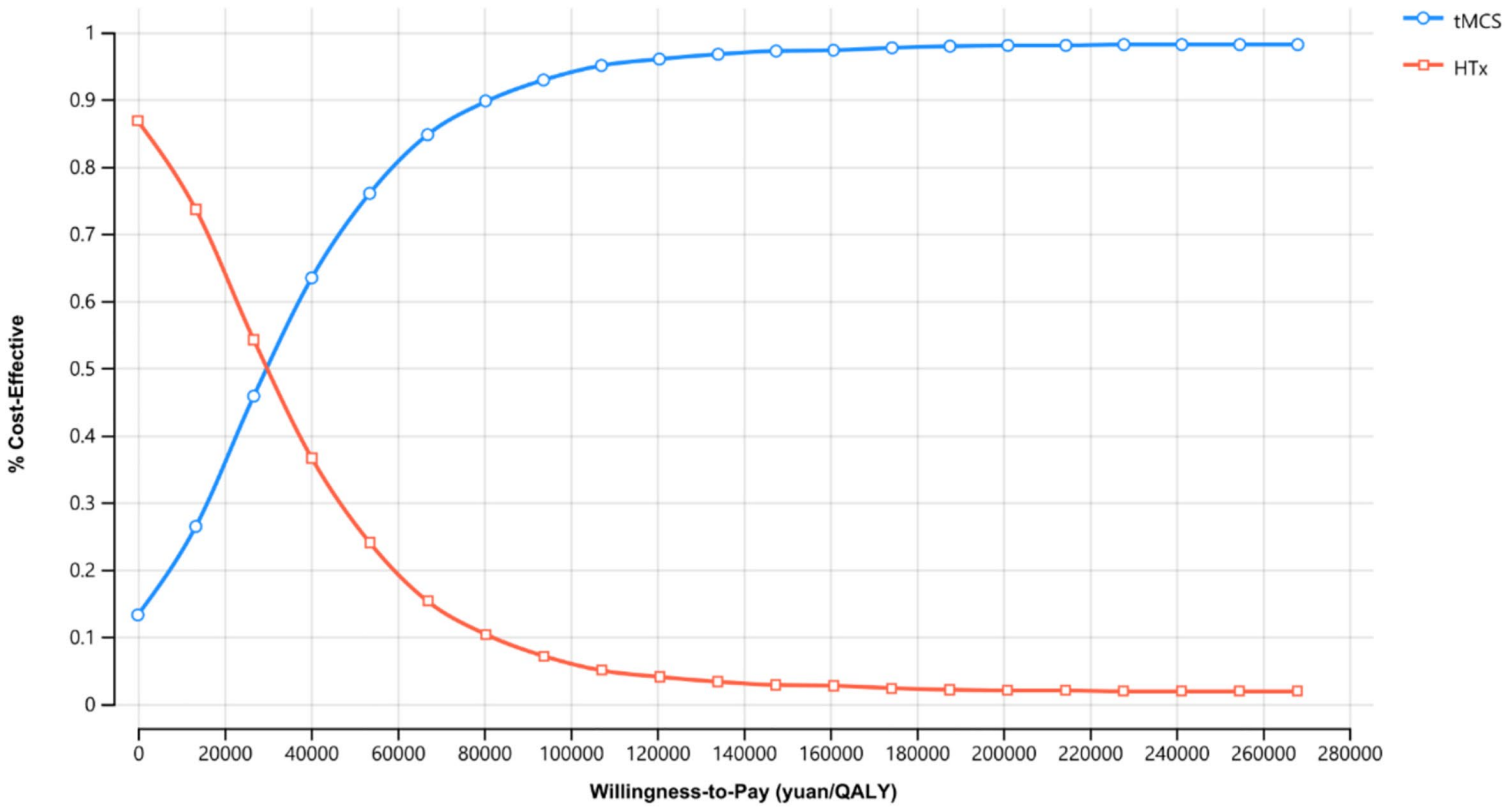
Costeffectiveness acceptability curves presenting the results of the probabilistic sensitivity analyses.

The univariate sensitivity analysis (Figure 1) indicated that, the model results were most sensitive to the utility values of heart transplant recipients receiving BTT-tMCS and those experiencing post-transplant infection. In the probabilistic sensitivity analysis, when the willingness to pay was >26807.40 RMB/QALY, the probability of BTT-tMCS being cost-effective was greater than that of non-bridged HTx patients; when the probability of cost-effectiveness reached 90%, the WTP for each QALY was 80422.20 RMB.

## Discussion

This study evaluated the cost-effectiveness of a bridge-to-transplantation temporary mechanical circulatory support (BTT-tMCS) for heart transplant patients in the Chinese population from the payer’s perspective and over a 10-year time horizon based on the Markov model. Propensity score matching was adopted to obtain 40 matched homogeneous heart transplant patients. This modeling analysis suggests that BTT-tMCS therapy in patients with heart transplant patients may represent a cost-effective treatment option generating additional quality-adjusted life years at an increased cost. The ICER point estimate was 28966.88 RMB/QALY, which can be considered an acceptable investment under recent threshold recommendations. The results were robust to parameter variation in sensitivity analyses (e.g., in the probabilistic sensitivity analysis, high certainty of cost-effectiveness was achieved at a willingness to pay of >26807.40 RMB/QALY).

According to the World Health Organization (WHO) recommendations, a health intervention is considered cost-effective if the incremental cost-effectiveness ratio (ICER) is below three times the gross domestic product (GDP) per capita of the country, and highly cost-effective if it is less than one times the GDP per capita. In 2023, China’s GDP per capita was approximately 89,358 RMB. The ICER for BTT-tMCS in our study was 28966.88 RMB/QALY, which is below the one-time GDP threshold, indicating that BTT-tMCS is highly cost-effective in the Chinese healthcare context.

In this study, the total cost of non-bridged HTx was 473,205 RMB, compared to 510,361 RMB for patients receiving BTT-tMCS therapy. Previous studies (29) have reported average heart transplant admission costs of 1704578.10 RMB in the United States (30), 1049729.20 RMB in Australia (31), 299938.80 RMB in Spain (32), and 419989.46 RMB in Brazil (33) (Using the online exchange rate of 1USD = 7.1884 Chinese RMBs, as of December 31, 2023). The total costs of heart transplantation in this study in China were 193705.72 RMB for non-bridged therapy and 277799.29 RMB for procedures involving BTT-tMCS. The comparison of the data shows that the hospitalization costs for heart transplantation in China are much lower than those in the USA and Australia, and closer to those in Spain and Brazil. The observed disparity in hospitalization costs may be attributed to a multitude of factors. It is possible that variations in healthcare system structures and reimbursement policies may be exerting a substantial influence on hospitalization expenses. Additionally, discrepancies in pricing for medical services and technologies could be contributing to the observed cost differences. Consequently, it is important to acknowledge that methodological variations, differences in publication timelines, and nuances in data collection strategies may limit the direct comparability of these findings with those of other studies.

The findings from the current study showed that the average quality-adjusted life years (QALYs) gained per capita with BTT-tMCS was 5.88, with an increase of 1.28 QALYs. This may be related to the clinical effect and actual health-related quality of life of preoperative mechanical circulatory support. Mechanical circulatory support as a bridge to transplantation has enabled patients with advanced heart failure to avoid progressive clinical deterioration while awaiting a suitable donor. A study in the United States showed that there was no difference in the in-hospital mortality rate after transplantation between patients who received tMCS and those who did not (15). The United Network for Organ Sharing (UNOS) study showed that, with the support of tMCS, HTx patients had better clinical outcomes, lower mortality rates, and shorter waiting times (12). These data support the use of tMCS before heart transplantation in appropriately selected patients. In addition, some patients may have temporary contraindications to transplantation, such as fixed elevated pulmonary vascular resistance or recent neoplasia (34). These patients must be bridged to transplantation by MCS to allow for sufficient end-organ recovery and physical rehabilitation before transplantation.

Besides, the effect in this study was measured by quality-adjusted life years (QALYs). The results of a cohort study in Germany showed that there were no significant differences in quality of life indicators, including mobility, self-care, daily activities, pain or discomfort, anxiety or depression, among survivors after HTx, regardless of whether preoperative mechanical circulatory support was performed or not (35). The heart transplant patients in this study were homogenized through propensity score matching and had similar clinical characteristics. Therefore, HTx survivors who underwent preoperative mechanical circulatory support may have a higher quality of life.

In the current study, over a 10-year long-term cycle, BTT-tMCS therapy had a higher cost-effectiveness compared with nonbridged HTx. This is consistent with the results of previous studies. A study in Canada showed that compared with direct heart transplantation, ventricular assist devices as a bridge to transplantation were more cost-effective (36). Multiple studies have evaluated the clinical effectiveness of left ventricular assist devices as a bridge to treatment (37, 38). While left ventricular assist devices offer benefits in terms of patient survival, functional status, and quality of life, studies suggest that non-bridged heart transplantation remains the preferred strategy for eligible patients, offering both prolonged survival and cost-effectiveness under most scenarios. Further reductions in adverse events or improvements in quality of life are needed for destination therapy-MCS to be cost-effective (39). Therefore, in the long term, tMCS has a higher cost-effectiveness, but it should be reasonably selected according to clinical circumstances (40).

We found that the utility of BTT-tMCS is an important factor affecting whether the BTT-tMCS therapy is higher cost-effective. When considering the effectiveness of BTT-tMCS therapy, the psychological impact of implanting mechanical circulatory support is an important factor that needs to be considered. Unfortunately, these values are inherently difficult to conceptualize. While it is easy to understand valuing one health state over another, attaching specific values to health states is more complex. Nevertheless, when the threshold set in this study is reached, when the willingness to pay is > 26807.40 RMB/QALY, the cost-effectiveness of BTT-tMCS is higher than that of non-bridged HTx.

Before and after PSM, the proportions of men and women participants converged. After PSM, the percentage of men was as high as 92.50%, women only 7.50% in the BTT-tMCS program, and 75.00% men and 25.00% women in the HTx program. This is consistent with previous studies: Women represent only about 25% of heart transplant recipients annually, who are less likely to be considered for heart transplantation (41–43). The sex subgroup analyses consistently demonstrate the superior cost-effectiveness of BTT-tMCS compared to HTx, irrespective of patient sex. This finding strongly supports the feasibility and potential value of implementing BTT-tMCS programs within the healthcare system. BTT-tMCS may represent a more efficient and equitable approach to heart transplantation, ensuring broader access to this life-saving intervention. Survival has improved for both women and men implanted with MCS.

In the BTT-tMCS regimen, males increased quality-adjusted life years at increased cost relative to females, resulting in higher cost-effectiveness. Evidences from multiple studies suggest that women who undergo preoperative mechanically circulation support (MCS) face poorer clinical outcomes. According to a US study, mortality in women was higher than in men (44). Cohort studies on left ventricular assist device (LVAD) showed a higher incidence rate of adverse events after LVAD implantation was also conducted seen in females compared with the males (45). In addition, reports have cited concerns for increased risk of stroke, major bleeding, need for RV support, and worse quality of life metrics in females (46). This suggests that future research should address the sex disparities in outcomes of contemporary MCS practices. In the nonbridged HTx regimen, male patients achieved greater quality-adjusted life years at increased cost compared with female patients, resulting in improved cost-effectiveness. Adversely, a review study suggested that women tend to have better long-term survival than men in post-transplantation (47). Few studies have systematically explored sex differences in post-HT outcomes (48). It warns us that we should strengthen our current understanding of gender differences in the field of HTx while identifying high-priority areas for future research.

In addition to demonstrating that the cost-effectiveness of BTT-tMCS, our findings have important implications for national policy and clinical decision-making. To facilitate integration into Chinese clinical guidelines, several actionable steps should be considered. First, national transplant committees and cardiovascular societies could incorporate cost-effectiveness thresholds into guideline development, identifying specific clinical scenarios where BTT-tMCS offers the greatest value such as in patients at high risk of waitlist deterioration (49, 50). Second, pilot implementation programs in high-volume transplant centers could test standardized protocols for BTT-tMCS initiation, data collection, and outcomes monitoring (51, 52). Third, policymakers may consider exploring conditional reimbursement schemes or tiered insurance coverage to improve access while managing financial risks (53). Finally, inclusion of BTT-tMCS outcomes in national transplant registries would support ongoing evaluation and evidence-based updates to clinical pathways (54).

This study has several limitations. First, although real-world cost and utility data were obtained from a Chinese transplant center, the majority of clinical transition probabilities used in the Markov model were sourced from international literature. These parameters may not fully reflect local treatment protocols, complication rates, or patient trajectories, which could affect model accuracy. Second, the analysis relied on a relatively small sample of 40 matched pairs (80 patients). While propensity score matching improved internal validity, the limited sample size reduces statistical power and may result in wider uncertainty intervals around ICER estimates. The single-center nature of the study further limits its external validity across different healthcare settings in China. Third, the Markov model used fixed annual cycles and assumed memoryless state transitions, which may oversimplify the disease progression and underestimate variability in patient outcomes. Future studies may consider discrete event simulation models that allow for greater flexibility and patient-level customization. Finally, this analysis was conducted solely from the healthcare payer’s perspective. It did not include patient out-of-pocket payments or societal costs like transportation, accommodation, productivity loss, home care, and frequent follow-up visits required for temporary MCS management. These costs may be substantial and should be included in future comprehensive evaluations.

## Conclusion

This study provides evidence that BTT-tMCS strategy may offer greater cost-effectiveness compared with nonbridged HTx in heart transplant recipients in Chinese population. In addition, this study highlights the critical need to deepen our understanding of gender-specific variations in heart transplantation (HTx) outcomes. Future research should prioritize elucidating the biological and psychosocial factors contributing to these observed differences and developing tailored interventions to optimize outcomes for both men and women.

## Data Availability

The raw data supporting the conclusions of this article will be made available by the authors, without undue reservation.
