# Supplementary material for: Cost-effectiveness analysis of bridge-to-transplantation temporary mechanical circulatory support versus non-bridged heart transplantation
Source: Front Public Health. 2026 Jan 8;13:1687327. doi: 10.3389/fpubh.2025.1687327 (PMC12823849; doi:10.3389/fpubh.2025.1687327)
Supplement: Supplementary file 1 [file Data_Sheet_1.docx]

**Supplemental Material**

Figure S1. The flowchart of participants selection of this study.

Figure S2. Model schematic.

Table S1. Costs and Utilities Imputed into the Model.

Figure S3. Balance test of PSM for tMCS and nonbridged HTx samples.

Figure S4. Markov state probability.

Figure S5. Cost-effectiveness plane.

Table S2. Transition probabilities by sex.

Table S3. Costs and Utilities by sex.

Figure S6. Tornado diagram of INMB.

A total of 162 participants from a qualified heart transplant center in central China

154 participants completed the questionnaire.

Participants were excluded if they were:

(1) missing, abnormal, or logically incorrect data;

(2) missing questionnaire information;

(3) fatal cases.

134 participants were included to analyze.

66 participants who were treated with BTT-tMCS were selected

68 participants who were treated with nonbridged-HTx were selected

80 participants were captured by 1:1 Propensity Score

Figure S1. The flowchart of participants selection of this study.

Survival

Survival

Dead

Dead

Re-implant MCS

BTT-tMCS therapy

Nonbridged HTx therapy

Figure S2. Model schematic.

Table S1 Costs and Utilities Imputed into the Model.

| Type | Model input parameters | Base case | Sensitivity analysis |
| --- | --- | --- | --- |
| Costs |  |  | Gamma: |
| BTT-tMCS | Survival  (Surgical hospitalization) | 277799.29 | 222,239.43-333,359.15 |
|  | Survival (>1 annually) | 15000.00 | 12000.00-18000.00 |
|  | Infection  (Surgical hospitalization) | 53485.89 | 42,788.71-64,183.07 |
|  | Infection (>1 annually) | 35000.00 | 28000.00-42000.00 |
|  | Renal failure  (Surgical hospitalization) | 103757.52 | 83,006.02-124,509.024 |
|  | Renal failure (>1 annually) | 40000.00 | 32000.00-48000.00 |
| HTx | Survival  (Surgical hospitalization) | 193705.72 | 154,964.58-232,446.86 |
|  | Survival (>1 annually) | 15000.00 | 12000.00-18000.00 |
|  | Infection  (Surgical hospitalization) | 246255.90 | 197,004.72-295,507.08 |
|  | Infection (>1 annually) | 35000.00 | 28000.00-42000.00 |
|  | Renal failure  (Surgical hospitalization) | 155032.98 | 124,026.38-186,039.58 |
|  | Renal failure (>1 annually) | 40000.00 | 32000.00-48000.00 |
|  | Implant MCS  (Surgical hospitalization) | 377,153.47 | 301,722.78-452,584.16 |
|  | Implant MCS (>1 annually) | 15000.00 | 12000.00-18000.00 |
| Utilities |  |  |  |
| BTT-tMCS | Survival | 0.956 | Beta:0.765-1.000 |
|  | Infection | 0.883 | Beta:0.706-1.000 |
|  | Renal failure | 0.873 | Beta:0.698-1.000 |
| HTx | Survival | 0.962 | Beta:0.770-1.000 |
|  | Infection | 0.566 | Beta:0.453-0.679 |
|  | Renal failure | 0.761 | Beta:0.609-0.913 |
|  | Implant MCS |  |  |
|  | Survival | 0.938 | Beta:0.750-1.000 |


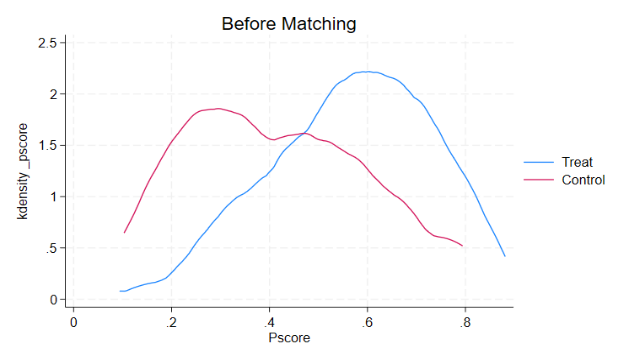

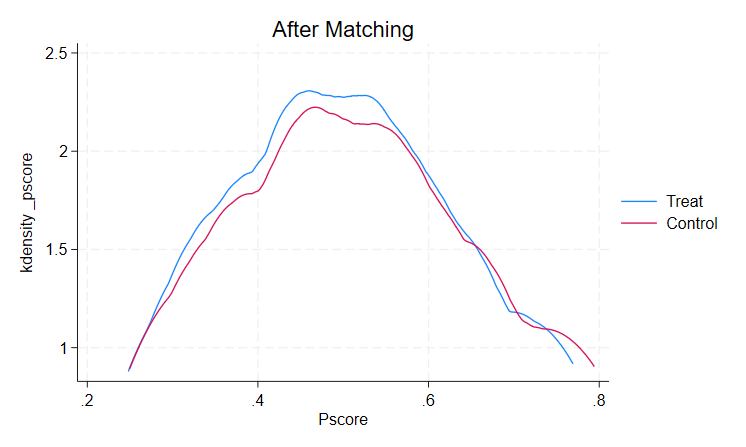


Figure S3. Balance test of PSM for tMCS and nonbridged HTx samples.


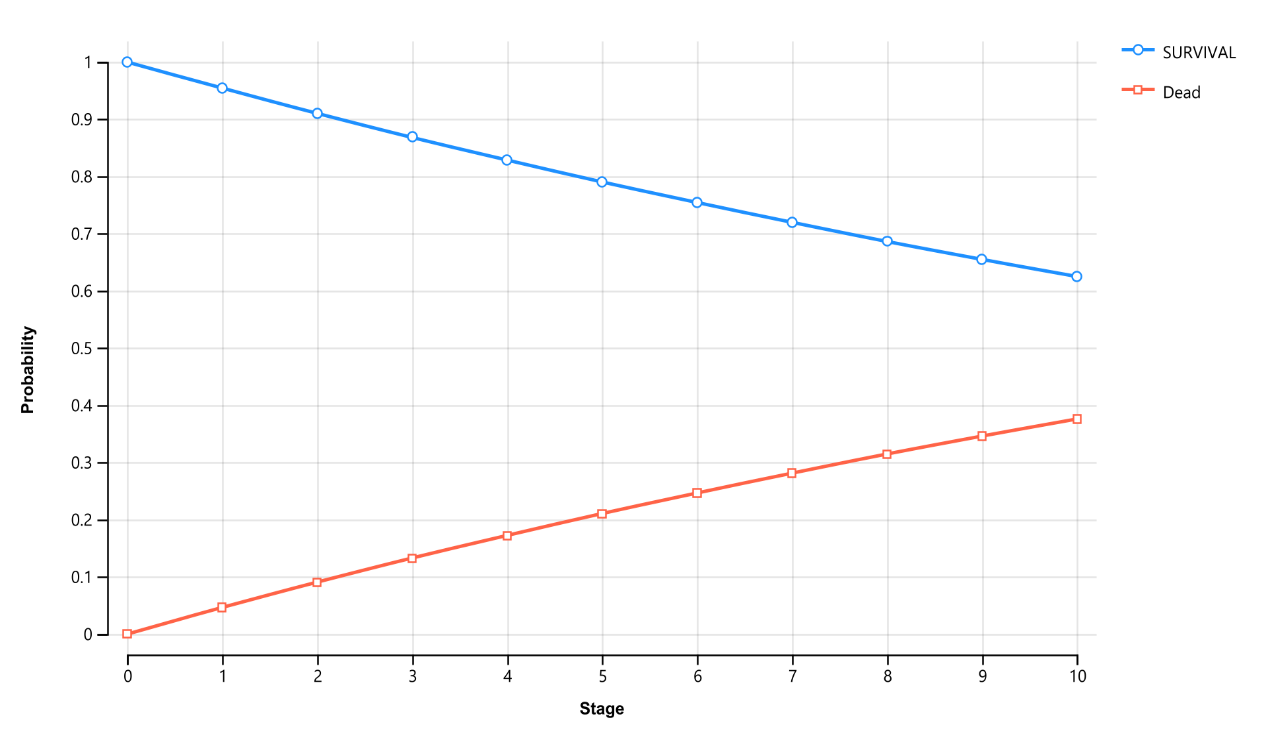


Figure S4. Markov state probability.


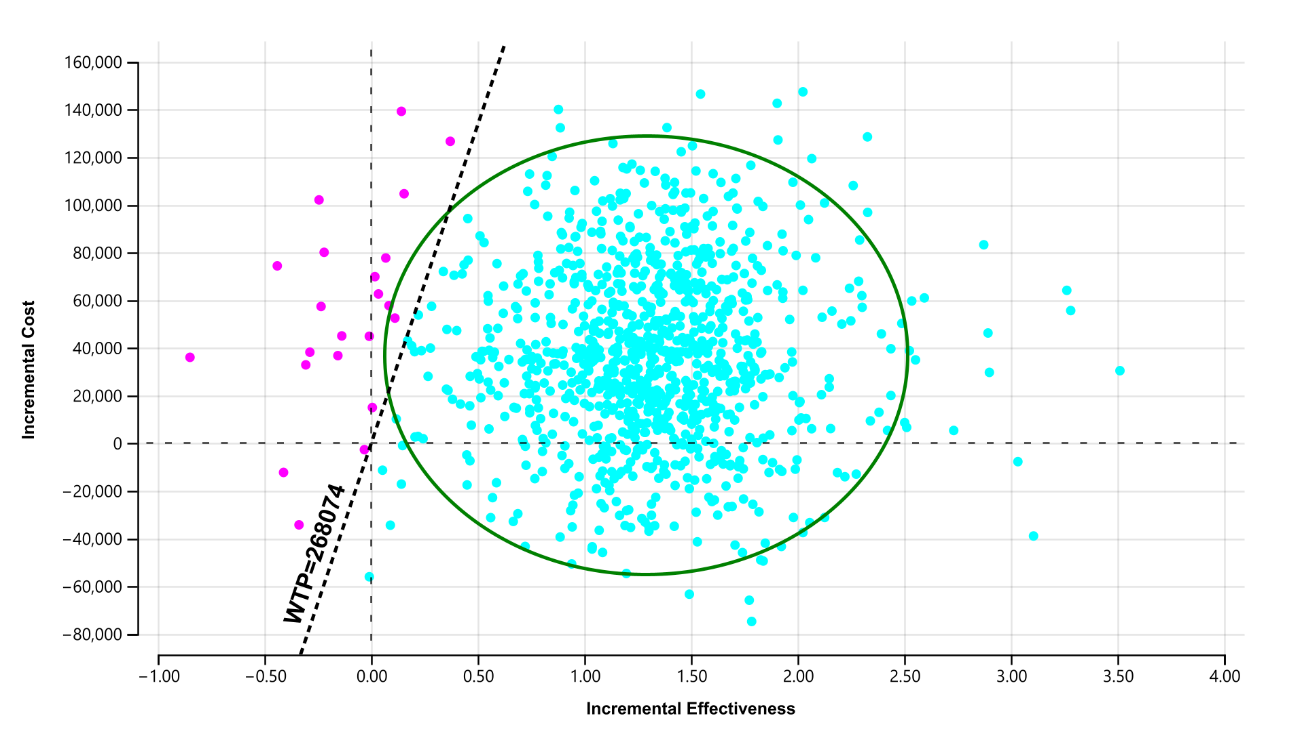


Figure S5. Cost-effectiveness plane. Scatterplot of effectiveness in quality-adjusted life years (QALYs) and costs for bridge-to-transplantation temporary mechanical circulatory support (BTT-tMCS) therapy versus nonbridged heart transplantation (HTx).

Table S2 Transition probabilities by sex.

|  | Male | | Female | |
| --- | --- | --- | --- | --- |
|  | Base case | Sensitivity analysis | Base case | Sensitivity analysis |
| BTT-tMCS |  |  |  |  |
| Infection | 0.307 | Beta: 0.293-0.322 | 0.333 | Beta: 0.304-0.361 |
| Renal acute | 0.278 | Beta: 0.263-0.292 | 0.252 | Beta: 0.225-0.279 |
| Dead | 0.137 | Beta: 0.126-0.148 | 0.147 | Beta: 0.125-0.169 |
| HTx |  |  |  |  |
| Infection | 0.340 | Beta: 0.307-0.372 | 0.342 | Beta: 0.280-0.404 |
| Infection  (>1 annually) | 0.307 | Beta: 0.293-0.322 | 0.333 | Beta: 0.304-0.361 |
| Renal acute | 0.244 | Beta: 0.215-0.274 | 0.224 | Beta: 0.169-0.278 |
| Renal acute  (>1 annually) | 0.278 | Beta: 0.263-0.292 | 0.252 | Beta: 0.225-0.279 |
| Implant MCS | 0.126 | Beta: 0.103-0.149 | 0.154 | Beta: 0.107-0.201 |
| Survival | 0.186 | Beta: 0.163-0.209 | 0.127 | Beta: 0.103-0.151 |
| Dead | 0.127 | Beta: 0.104-0.150 | 0.171 | Beta: 0.122-0.221 |

Table S3 Costs and Utilities by sex.

| Type | Therapy | Male | | Female | |
| --- | --- | --- | --- | --- | --- |
|  |  | Base case | Sensitivity analysis | Base case | Sensitivity analysis |
| Cost | BTT-tMCS |  |  |  |  |
|  | Survival | 280,755.00 | Gamma:  224,604-  336,906 | 195,039.50 | Gamma:  156,031.6-234,047.4 |
|  | Infection | 17,091.04 | Gamma:  13,672.83-20,509.25 | 167,275.44 | Gamma:  133,820.35-200,730.53 |
|  | Renal acute | 30,443.59 | Gamma:  24,354.87-36,532.31 | 608,666.66 | Gamma:  486,933.33-730,399.99 |
|  | HTx |  |  |  |  |
|  | Survival | 195,604.46 | Gamma:  156,483.57-234,725.35 | 188,009.50 | Gamma:  150,407.6-225,611.4 |
|  | Infection | 402,500.81 | Gamma:  322,000.65-483,000.97 | 93,808.46 | Gamma:  75,046.77-112,570.15 |
|  | Renal acute | 186,594.81 | Gamma:  149,275.69-223,913.53 | 93,808.46 | Gamma:  75,046.77-112,570.15 |
|  | Implant MCS |  |  |  |  |
|  | Survival | 438,888.84 | Gamma:  351,111.07-526,666.61 | 517457.11 | Gamma:  413,965.69-620,948.53 |
|  | Annually |  |  |  |  |
|  | Survival | 15000.00 | Gamma:  12000.00-18000.00 | 15000.00 | Gamma:  12000.00-18000.00 |
|  | Infection | 35000.00 | Gamma:  28000.00-42000.00 | 35000.00 | Gamma:  28000.00-42000.00 |
|  | Renal failure | 40000.00 | Gamma:  32000.00-48000.00 | 40000.00 | Gamma:  32000.00-48000.00 |
| Utility | BTT-tMCS |  |  |  |  |
|  | Survival | 0.956 | Beta:0.765-1.000 | 0.951 | Beta:0.761-1.000 |
|  | Infection | 0.843 | Beta:0.674-1.000 | 0.893 | Beta:0.714-1.000 |
|  | Renal acute | 0.867 | Beta:0.694-1.000 | 0.907 | Beta:0.726-1.000 |
|  | HTx |  |  |  |  |
|  | Survival | 0.955 | Beta:0.764-1.000 | 0.982 | Beta:0.786-1.000 |
|  | Infection | 0.412 | Beta:0.330-0.494 | 0.720 | Beta:0.576-0.864 |
|  | Renal acute | 0.781 | Beta:0.625-0.937 | 0.720 | Beta:0.576-0.864 |
|  | Implant MCS | 0.947 | Beta:0.758-1.000 | 0.907 | Beta:0.726-1.000 |

A

B

C


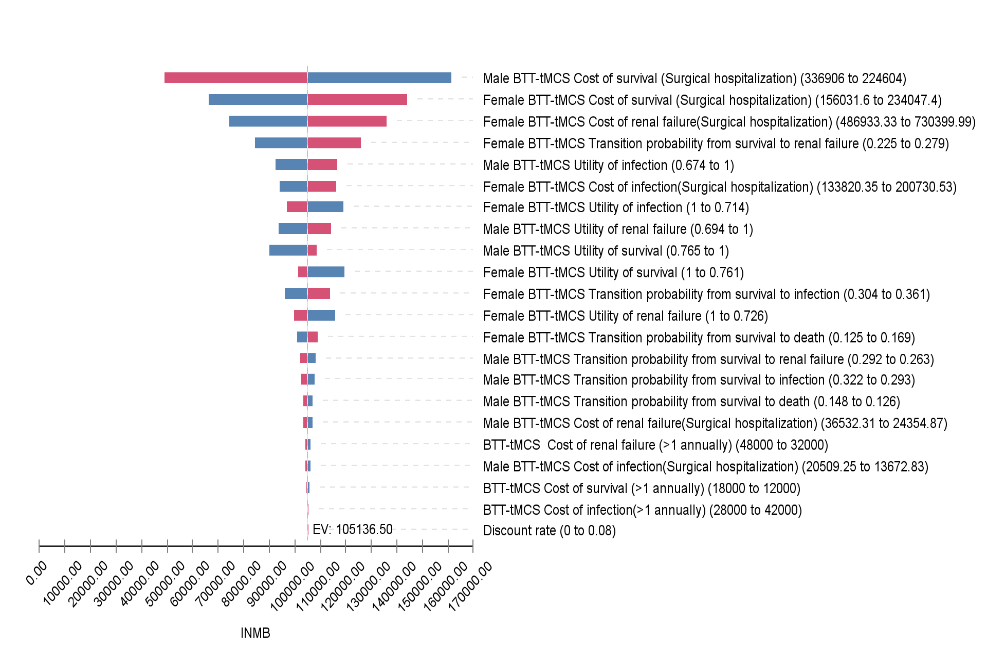

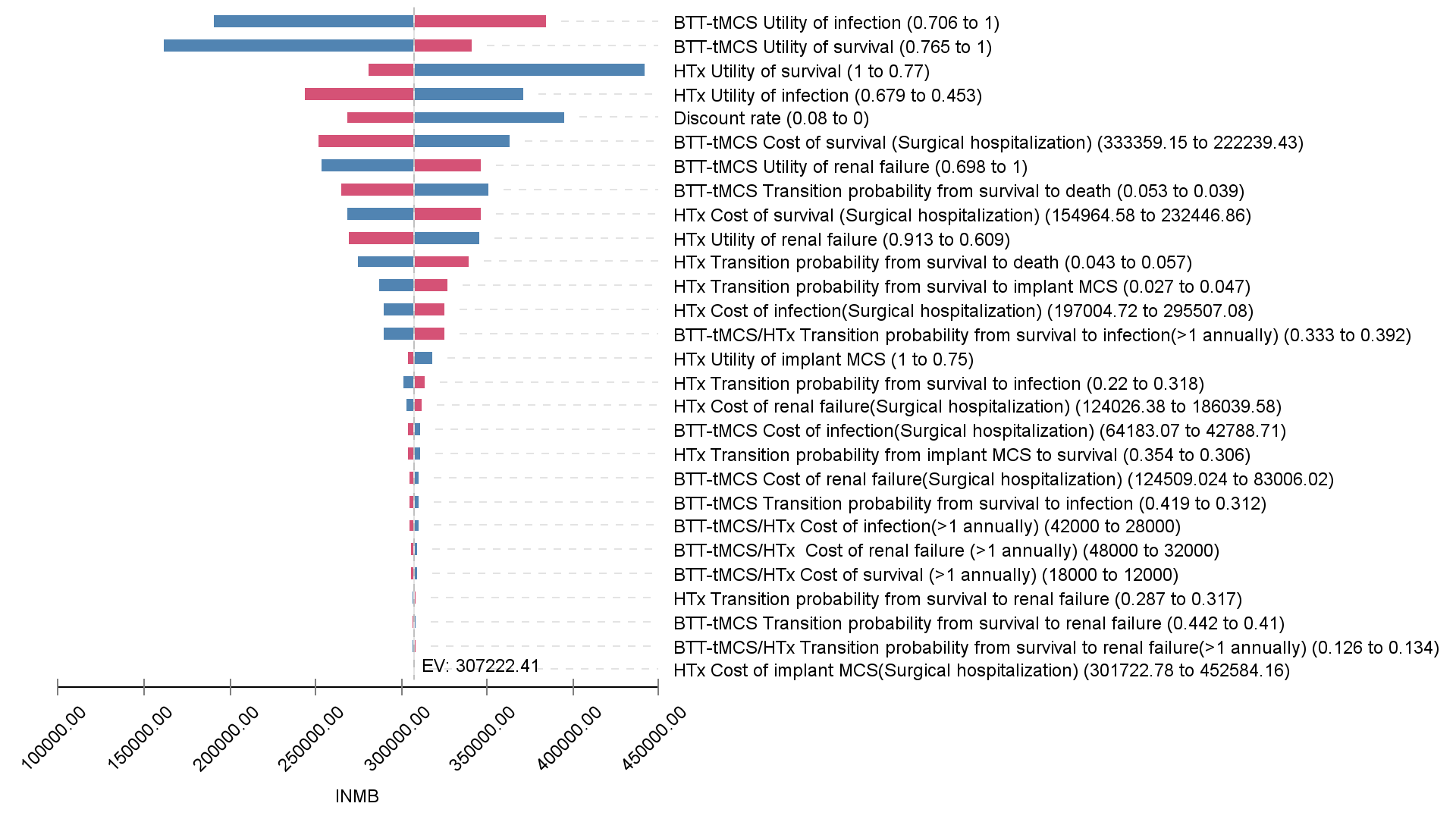

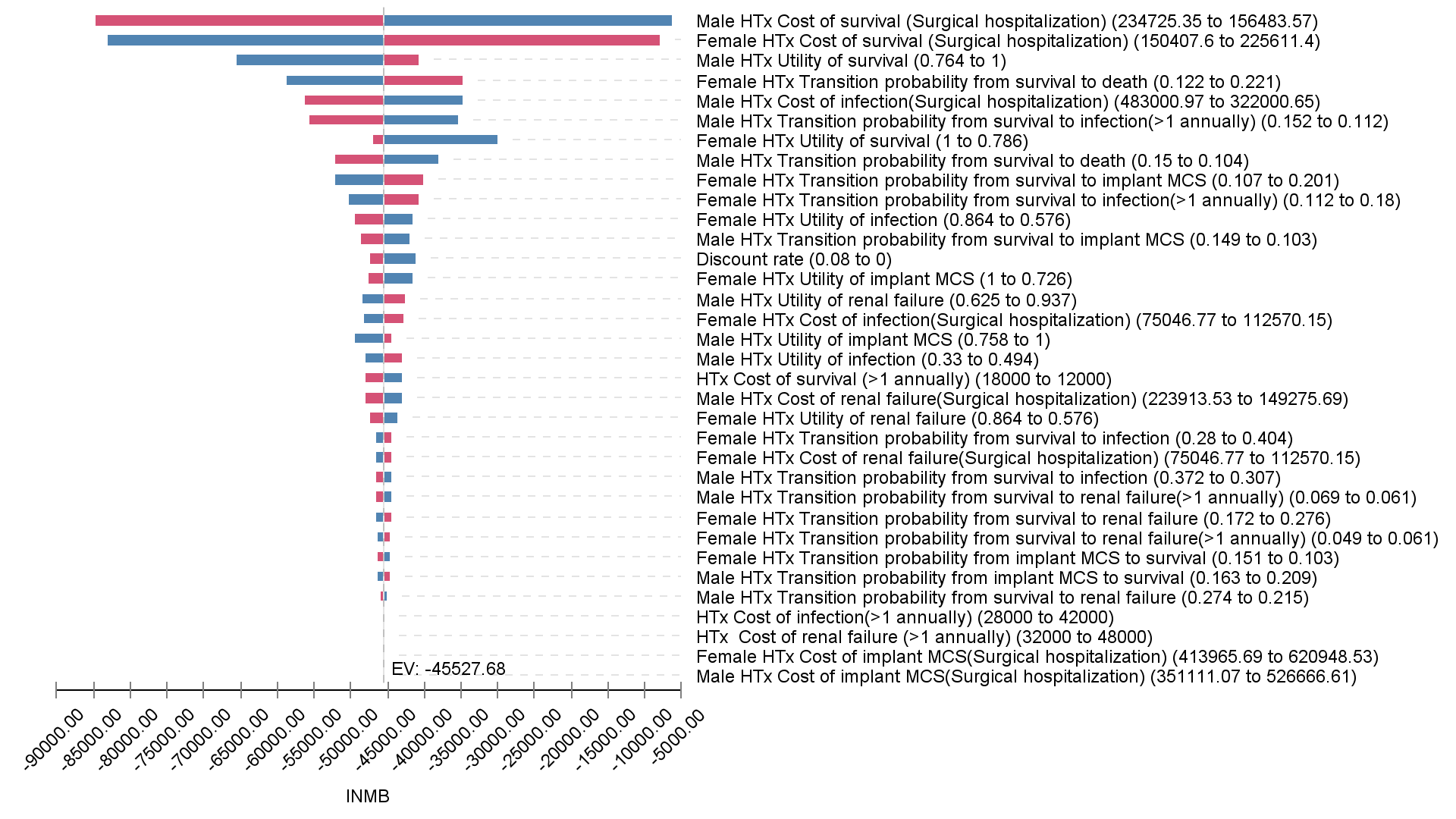


Figure S6. Tornado diagram of INMB(Incremental Net Monetary Benefit) showing the changes in BTT-tMCS cost-effectiveness relative to non-bridged HTx of the deterministic sensitivity analysis at 10-year time horizon. (A) BTT-tMCS and HTx two therapies (B) Male and female subgroups in tMCS therapy (C) Male and female subgroups in HTx therapy**.** Blue bars: effectiveness of decreasing the parameter value, red bars: effectiveness of increasing the parameter value.
